# Things to keep in mind when selecting physical assessments in youth soccer: Correlations between test performances, interlimb asymmetries, and effects of maturation

**DOI:** 10.1371/journal.pone.0305570

**Published:** 2024-06-21

**Authors:** Francisco Javier Robles-Palazón, John J. McMahon, Francisco Ayala, Paul Comfort

**Affiliations:** 1 Department of Physical Activity and Sport, Faculty of Sport Sciences, Campus of Excellence Mare Nostrum, University of Murcia, Murcia, Spain; 2 Centre for Human Movement and Rehabilitation, University of Salford, Salford, United Kingdom; 3 School of Sport and Exercise, University of Gloucestershire, Gloucester, United Kingdom; 4 School of Medical and Health Sciences, Edith Cowan University, Joondalup, Australia; Universidad de León Facultad de la Ciencias de la Actividad Física y el Deporte: Universidad de Leon Facultad de la Ciencias de la Actividad Fisica y el Deporte, SPAIN

## Abstract

A range of field-based tests have been proposed for inclusion in physical performance assessment batteries. However, there are obvious time and human resources constraints in applied settings. The knowledge of potential relationships between tests on performance, asymmetries, as well as maturation-induced changes, may help select the most informative and least time-consuming testing battery. The purposes of this study were: (1) to determine correlations in performance between different field-based tests, in interlimb asymmetry between those unilateral tests, and between asymmetry scores and test performances, and (2) to determine the influence of players’ maturity status on test performances and asymmetries. A total of 309 male youth soccer players completed a nine-test battery including y-balance test, drop vertical jump, countermovement jump, single leg countermovement jump, standing long jump, single leg hop for distance, Illinois agility test, 10 m sprint, and 20 m sprint. The results revealed moderate-to-very large relationships between jump, sprint, and agility performances (*r* = 0.43–0.94), but weak-to-moderate correlations between these tests and balance scores (*r* ≤ 0.38). No relevant relationship (*r* ≤ 0.32) for asymmetries detected through different unilateral tests was found, nor between asymmetries and performance scores (*r* < 0.29). While maturity status clearly influenced players’ performance, a limited impact on asymmetries was evident. Despite the mentioned relationships, the low shared variance between tests indicates that they should not be used interchangeably, and coaches should select those with the greatest specificity for the sport. Asymmetries do not influence performance, but their prevalence and unchanging nature with maturation can be seen as an opportunity for the identification of highly asymmetrical players and the application of interventions to improve the weaker limb, irrespective of the athlete’s stage of development. To this end, the single leg countermovement jump might be viewed as an appropriate test in male youth soccer.

## 1. Introduction

Young athletes participating in physically demanding sports such as soccer are required to perform multiple repeated high-intensity and multidirectional actions (e.g., jumping, sprinting, and change of direction maneuvers) during training and matches [1]. The ability to perform these actions has been identified as an important prerequisite for successful participation in team sports [2] and thus, a range of field-based tests have been proposed for inclusion in physical performance assessment batteries [3, 4]. However, there are obvious time and human resources constraints when a large number of players must be screened in applied settings. The inclusion of multiple tests may then not always be possible, and the knowledge of potential relationships between test performances may help coaches to select the most informative and least time-consuming testing battery.

Previously researchers have reported large to very large relationships between different physical performance abilities, such as vertical jump height and linear sprint time [5–9]. Conversely, others have failed to reach these high levels of correlations between the same physical attributes [10–13]. Several factors have been proposed as responsible for these observed discrepancies, including participants’ age, sex, sport, and level of performance, but also the use of small sample sizes, and the application of different methodologies and limited number of tests to evaluate these characteristics [13, 14]. Although associations among physical tests frequently implemented in youth soccer environments have been examined in some previous studies [7, 15–17], researchers using an extended approach to analyze both the correlation between tests assessing different physical performance abilities (e.g., sprint vs. jump) and the interrelationships between tests (e.g., 10 m vs. 20 m sprints, countermovement vs. drop jumps) involving the same movement pattern (i.e., linear sprint, vertical jump) in a large sample of male youth soccer players are scarce [7, 17]. This extended approach can guide practitioners’ physical assessment selection; for example, if one jump test can explain performance in other jump assessments and different abilities such as sprinting, then the test battery may be logically reduced. Thus, more comprehensive research concurrently exploring the correlation between different abilities and using multiple tests is needed in this population.

The analysis of interlimb asymmetries has also been another major goal of physical testing batteries [3, 4]. The available body of evidence indicates that interlimb asymmetries across a range of unilateral tests [18–20] are prevalent in youth soccer players. The magnitude of asymmetries (percentage of difference between limbs) is test-specific, with researchers reporting variations in average inter-limb difference ranging from 6–7% to 12% for key performance outcomes (i.e., distance and height) in different horizontal and vertical jump tests, respectively [19–21]. Likewise, a variation in the direction (right vs. left limb) of asymmetry across tests has also been reported in youth soccer players [20, 22–24], with the inclusion of multiple assessments recommended when assessing asymmetries in youth athletes [3, 25]. Despite these findings, the existing evidence regarding the consistency of asymmetry values (and its direction) in male youth soccer players is mainly limited to vertical jump tests evaluated in small cohorts (< 50 athletes) [22–24]. Again, the study of the potential relationships between unilateral tests may help to optimize screening batteries.

However, it should be noted that asymmetries may simply be a by-product of playing competitive sport over time [26], and their correction should only be prioritized where these interlimb differences represent a significant risk factor for injury and/or impair the young athlete’s performance [18]. While some researchers have identified a link between asymmetries and increased injury risk [27, 28], their influence on athletic performance is conflicting. For example, Lockie et al. [29] did not find any relationships (*r* < 0.19; *p* > 0.05) between interlimb jump asymmetries and performance in multidirectional speed tests in a cohort of recreational team-sport (including soccer) athletes; on the contrary, Bishop et al. [18] found strong correlations (*r* = 0.51–0.87; *p* < 0.05) between interlimb jump asymmetries and youth soccer players’ performance in jumping and multidirectional speed tests.

In male youth athletes, physical performance has been shown to generally increase with chronological age [7, 30]. These differences across age groups have frequently been attributed to maturation [7, 30], and recent evidence has confirmed this suggested interaction between physical performance (e.g., sprint [31], jump [32]) and maturity status in adolescent soccer players. Nevertheless, the development of certain physical performance abilities (e.g., balance) across maturity states is still unclear [33, 34]. Likewise, a few studies have explored the influence of maturation on interlimb asymmetries, reporting a limited impact of stage of maturation on mean asymmetry values [19, 35]. However, a large interplayer variability in asymmetry scores has also been detected across several unilateral tests [25] and as such reporting only group average may have distorted the true effect of maturation on interlimb asymmetry. Consequently, additional studies are required to fully explore maturation-related changes in physical performance and asymmetry scores, while analyzing side-to-side differences on a more individual level [25].

The purposes of this study were: (1) to determine the correlations in players’ performance for different field-based tests, in interlimb asymmetry between those unilateral tests, and between asymmetry scores and test performances, and (2) to determine the influence of players’ maturity status on test performance and asymmetry results. Based on previous research, it was hypothesized that: (1) at least moderate relationships would be found between multidirectional sprint and jump tests scores [7, 17], while weak correlations were expected for asymmetries detected through different tests and between asymmetries and test performances [25, 29]; and (2) players who were more mature would have greater scores for test performances [31, 32], but differences between maturational stages would be relatively small for asymmetries [19, 35].

## 2. Materials and methods

### 2.1 Study design

A cross-sectional observational design was used. Participants attended their respective club’s training facilities during the preseason period of the competitive years 2017/18 and 2018/19 (i.e., from August 28 to September 15, 2017, and from August 27 to September 14, 2018), and all data were collected in a single testing session for each soccer team. Anthropometric measures needed to calculate the maturity status were recorded first, and then the players performed different neuromuscular tests including dynamic balance, vertical and horizontal jumps, agility (change of direction), and sprint. A 20-minute standardized dynamic warm-up was completed before the performance tests, which included whole body exercises emphasizing dynamic mobilization and gradually progressing in intensity [36].

### 2.2 Participants

A total of 309 male youth (10–19 years old) soccer players volunteered to participate in this study (Table 1). Participants met the following inclusion/exclusion criteria: 1) engaged regularly in soccer (at least 2–3 trainings and 1 match per week), and 2) were free of injuries and delayed onset muscle soreness at the time of testing (self-reported). Participants were asked to refrain from vigorous exercise at least 48 hours prior to the testing session. The experimental procedures used were in accordance with the Declaration of Helsinki and were approved by the Ethics and Scientific Committee of the University of Murcia (ID: 1551/2017). Likewise, written informed consent and assent was obtained from participants and parents/legal guardians.

**Table 1:** Descriptive anthropometric values (mean ± standard deviation) for all the participants and per maturation group.

Group	*n*	Age (years)	Body mass (kg)	Stature (cm)	Leg length (cm)	Maturity offset
Pre-PHV	118	11.6 ± 0.9	40.9 ± 7.2	149.2 ± 7.0	73.5 ± 4.8	−2.2 ± 0.7
Circa-PHV^a^	44	13.9 ± 0.7	57.4 ± 7.1	167.5 ± 4.8	82.9 ± 4.5	0.0 ± 0.3
Post-PHV	100	16.6 ± 1.3	67.8 ± 8.0	177.0 ± 6.2	86.6 ± 5.0	2.2 ± 0.8
Whole group	309	13.9 ± 2.4	54.0 ± 13.6	163.2 ± 13.4	80.4 ± 7.4	−0.2 ± 2.0

^a^ To account for the reported error in the prediction equation used to calculate players’ maturity offset, participants (*n* = 47) with years from age at peak height velocity (PHV) ranging from −1 to −0.5 and from 0.5 to 1 were removed. Thus, the circa-PHV group consisted of players showing a maturity offset from −0.5 to 0.5.

### 2.3 Procedures

#### 2.3.1 Anthropometry and maturity status

Body mass was measured on a calibrated physician scale (SECA 799, Hamburg, Germany). Standing and sitting heights were recorded on a measurement platform (SECA 799, Hamburg, Germany). Players’ leg length was calculated as the difference between their recorded standing and sitting heights. Maturity status was calculated using the equation of Mirwald et al. [37]. To account for the reported error (approximately 6 months) in this equation, players were grouped into discrete bands based on their maturational offset (pre-PHV [<-1], circa-PHV [-0.5 to 0.5], post-PHV [>1]), and players with a maturity offset from -1 to -0.5 and 0.5 to 1 were removed (*n* = 47) from the dataset when players were analyzed by stage of maturation.

#### 2.3.2 Neuromuscular performance assessment

The nine tests were concurrently performed using a randomized circuit style approach: y-balance test (YBT), drop vertical jump (DVJ), countermovement jump (CMJ), single leg countermovement jump (SLCMJ), standing long jump (SLJ), single leg hop for distance (SLHD), Illinois agility test, 10 m sprint, and 20 m sprint. Tests were selected based on their suitability and reliability for use within the context of youth soccer [3, 18, 20, 33, 38]. All measures were taken by trained testers (i.e., Masters and PhD students in Sports Science) coordinated by the principal investigator (FJR-P). Dominant leg was defined as the player’s preferred kicking leg.

*2*.*3*.*2*.*1 Y-balance test (YBT)*. Participants placed their hands on their hips and began in a unilateral stance with the most distal aspect of their great toe behind the line on the center of the Y-Balance test kit^™^ (Functional Movement Systems, USA). They were then asked to reach the maximal distance by pushing the target indicator in the anterior, posteromedial, and posterolateral directions, while maintaining their unilateral stance. Specifically, testing order was completed as dominant anterior, non-dominant anterior, dominant posteromedial, non-dominant posteromedial, dominant posterolateral, and non-dominant posterolateral. Trials were discarded if the player failed to maintain unilateral stance on the platform, failed to maintain reach foot contact with the reach indicator on the target area while the reach indicator is in motion, used the reach indicator for stance support, or failed to return the reach foot to the starting position under control [39]. After three familiarization repetitions, three trials were performed on each leg and for each direction. The reached distance in cm was recorded and was then normalized and analyzed as percentage of leg length (excursion distance / leg length × 100 = % of leg length) [39]. A composite score (average of the three directions) was also calculated to obtain a global measure of the balance performance.

*2*.*3*.*2*.*2 Drop vertical jump (DVJ)*. Participants stood with hands on hips and feet shoulder-width apart on a 40 cm high box [40]. They were instructed to lean forward and drop from the box. Players were required to land with both feet simultaneously on a contact mat (Ergo Jump Bosco System, Italia) that was located 20 cm in front of the box, then immediately perform a maximal vertical jump minimizing ground contact time, and finally land back on the contact mat. After three familiarization repetitions, each player performed two maximal jumps. Jump height was used for analysis.

*2*.*3*.*2*.*3 Countermovement jump (CMJ)*. Bilateral CMJs were carried out on a contact mat (Ergo Jump Bosco System, Italia). Participants stood with hands on hips and performed a countermovement to a self-selected depth, before jumping as high as possible and landing back on the contact mat. After three familiarization repetitions, each player performed two maximal trials. Jump height was obtained for analysis.

*2*.*3*.*2*.*4 Single leg countermovement jump (SLCMJ)*. SLCMJs were performed with dominant and non-dominant legs. Participants stood on the test leg in the center of a contact mat (Ergo Jump Bosco System, Italia) with hands on hips and the knee of the non-jumping leg slightly flexed, so that the hovering foot was positioned at approximately mid-shin height of the jumping leg. Players performed a countermovement to a self-selected depth, before jumping as fast as possible and landing on the same test leg. No swinging of the non-jumping leg was allowed. After three familiarization repetitions, each player performed two maximal trials on each leg. Performance leg was alternated until all trials were completed. Jump height was used for analysis.

*2*.*3*.*2*.*5 Standing long jump (SLJ)*. Participants stood behind the starting line and were instructed to push off vigorously and jump as far as possible. They had to land with the feet together and to stay upright. Free movement of the arms was allowed during the test. Jump distance was measured from the starting line to the player’s heel with a standard tape measure. After two familiarization tests, participants performed three repetitions.

*2*.*3*.*2*.*6 Single leg hop for distance (SLHD)*. Participants stood behind the starting line on a unilateral stance and were instructed to hop as far as possible, landing on the same leg. To be considered a successful trial, participants must land under complete control, holding the final landing for at least 2 s. Trials were conducted with dominant and non-dominant legs, and performance leg was alternated until all trials were completed. After two practice trials, participants performed three maximal repetitions on each leg. The hop distance in cm was recorded and was then normalized and analyzed as percentage of leg length (hop distance / leg length × 100 = % of leg length) [32].

*2*.*3*.*2*.*7 Illinois agility test*. The Illinois agility test set-up consists of an area measuring 10 m in length and 5 m in width (distance between the start and finish points). Four cones are used to mark the start and finish lines as well as the two corners where participants turn. Four cones are placed in the center of the testing area at a distance of 3.3 m from one another. To complete the test, participants sprinted 10 m from the start position to the first corner cone, turned to weave down and back through the center line of cones, made one final change of direction at the second corner cone and finished with another 10-m sprint to the finish line. Athletes were instructed to complete the trial as fast as possible without knocking down any cones. After a familiarization test, participants performed two maximal trials. Time was measured using a photocell system (Microgate Witty photocells; Microgate, Italy).

*2*.*3*.*2*.*8 10 m and 20 m sprint*. Time during a 10 m and 20 m sprint in a straight line was measured by means of three pairs of Microgate Witty photocells (Microgate, Italy) placed 1.0 m above the ground level. Each sprint was initiated from an individually chosen standing position, 50 cm behind the photocell gate, which started a digital timer. After a familiarization test, participants performed two maximal trials.

### 2.4 Statistical analyses

Raw data sets were checked for normality using the Kolmogorov-Smirnov test. Descriptive statistics including means and standard deviations (SDs) were calculated for all measures.

Pearson’s product-moment correlation coefficient (*r*) was used to determine (1) linear correlations in performance between all tests, (2) the relationship in interlimb asymmetry between unilateral tests, and (3) the association between asymmetry scores and test performance. Correlation coefficients were interpreted as follows: ≤0.10, trivial; 0.11–0.30, small; 0.31–0.50, moderate; 0.51–0.70, large; 0.71–0.90, very large; >0.90, almost perfect [41]. The Bonferroni method was used to adjust *p*-values for family-wise error rate.

A one-way ANOVA determined between-group differences for test performance and asymmetries by maturity status (pre-PHV, circa-PHV, post-PHV). Homogeneity of variance was tested by Levene’s statistic and, where violated, Brown-Forsythe adjustment was used. Omega-squared (*ω*^*2*^) effect size (ES) was calculated for all main effects between maturity groups, and was interpreted as follows: ≥0.01, small; ≥0.06, medium; ≥0.14, large [42]. Post hoc comparisons were made to determine significant pairwise differences using the Bonferroni or Games-Howell test when equal variance was or was not assumed, respectively. Cohen’s *d* ES was also calculated for all pairwise contrasts, considering *d* ≥0.2 small, ≥0.5 medium, and ≥0.8 large [43].

To analyze the correlation in asymmetry between unilateral tests, the interlimb absolute asymmetry was computed ((dominant—nondominant)) / (max: dominant or nondominant) × 100) [44], and thus the direction of asymmetries was considered (i.e., negative and positive values representing asymmetries in favour of nondominant and dominant legs, respectively). To determine the association between asymmetry scores and test performances, as well as to identify potential differences by maturity status, the bilateral strength asymmetry equation was applied ((stronger—weaker)) / (stronger) × 100), and only the standard percentage difference between legs was used for these analyses [44].

The proportion of players showing large asymmetries across maturation groups was also explored. The cut-off value of ≥10% was selected to determine large asymmetries [27]. Kruskal-Wallis test was used to examine these differences across maturation groups, and pairwise comparisons were determined through the Dunn’s post hoc test using the Bonferroni correction.

All analyses were performed with JASP computer software (version 0.16.4.0; JASP, Amsterdam, The Netherlands), with the significance level set at *p* < 0.05 for all tests.

## 3. Results

### 3.1 Correlations between tests

Pearson’s correlations showed moderate-to-almost perfect relationships (*r* = 0.43 to 0.94; *p* < 0.05) between all the different test performances, with the exception of the YBT measures which, apart from the moderate-to-very large relationships (*r* = 0.41 to 0.90; *p* < 0.05) found between its different YBT directions, only demonstrated moderate associations (*r* = 0.33 to 0.38; *p* < 0.05) for YBT-PM, YBT-PL, and YBT-CS with the SLHD. All jump tests showed large-to-very large correlations (*r* = 0.55 to 0.81; *p* < 0.05), with the highest *r*-values being found between tests assessing the same jump direction (vertical vs. horizontal). 10 m and 20 m sprint tests were strongly correlated (*r* = 0.94; *p* < 0.05), and both sprint tests also exhibited a very large correlation (*r* = 0.72; *p* < 0.05) with Illinois agility test times (Table 2).

**Table 2:** Correlations in performance scores between the different tests.

**Test**		**YBT-A (%)**	**YBT-PM (%)**	**YBT-PL (%)**	**YBT-CS (%)**	**DVJ (cm)**	**CMJ (cm)**	**SLCMJ (cm)**	**SLJ (cm)**	**SLHD (%)**	**Illinois agility (s)**	**10 m sprint (s)**	**20 m sprint (s)**
YBT-A (%)	*r*	—											
	(95% CI)												
YBT-PM (%)	*r*	0.41*	—										
	(95% CI)	(0.31 to 0.50)											
YBT-PL (%)	*r*	0.48*	0.71*	—									
	(95% CI)	(0.38 to 0.56)	(0.65 to 0.76)										
YBT-CS (%)	*r*	0.69*	0.88*	0.90*	—								
	(95% CI)	(0.63 to 0.75)	(0.85 to 0.90)	(0.88 to 0.92)									
DVJ (cm)	*r*	−0.06	0.18*	0.09	0.10	—							
	(95% CI)	(−0.18 to 0.06)	(0.06 to 0.29)	(−0.03 to 0.21)	(−0.02 to 0.22)								
CMJ (cm)	*r*	−0.04	0.21*	0.19*	0.17	0.79*	—						
	(95% CI)	(−0.16 to 0.09)	(0.08 to 0.32)	(0.07 to 0.31)	(0.04 to 0.28)	(0.74 to 0.83)							
SLCMJ (cm)	*r*	−0.11	0.22*	0.16	0.13	0.69*	0.77*	—					
	(95% CI)	(−0.24 to 0.01)	(0.10 to 0.34)	(0.03 to 0.28)	(0.01 to 0.26)	(0.61 to 0.75)	(0.70 to 0.82)						
SLJ (cm)	*r*	−0.05	0.26*	0.20*	0.19*	0.59*	0.66*	0.68*	—				
	(95% CI)	(−0.16 to 0.07)	(0.15 to 0.36)	(0.09 to 0.31)	(0.08 to 0.30)	(0.51 to 0.66)	(0.58 to 0.72)	(0.60 to 0.74)					
SLHD (%)	*r*	0.04	0.38*	0.33*	0.33*	0.55*	0.59*	0.63*	0.81*	—			
	(95% CI)	(−0.07 to 0.16)	(0.28 to 0.48)	(0.22 to 0.43)	(0.22 to 0.43)	(0.46 to 0.63)	(0.50 to 0.67)	(0.55 to 0.70)	(0.76 to 0.85)				
Illinois agility (s)	*r*	−0.10	−0.26*	−0.24*	−0.25*	−0.44*	−0.43*	−0.57*	−0.52*	−0.54*	—		
	(95% CI)	(−0.22 to 0.02)	(−0.37 to −0.15)	(−0.35 to −0.12)	(−0.36 to −0.13)	(−0.54 to −0.33)	(−0.53 to −0.32)	(−0.66 to −0.47)	(−0.60 to −0.42)	(−0.62 to −0.44)			
10 m sprint (s)	*r*	0.05	−0.18*	−0.16	−0.14	−0.59*	−0.56*	−0.72*	−0.62*	−0.59*	0.72*	—	
	(95% CI)	(−0.06 to 0.17)	(−0.29 to −0.07)	(−0.27 to −0.05)	(−0.25 to −0.02)	(−0.66 to −0.50)	(−0.64 to −0.47)	(−0.78 to −0.65)	(−0.69 to −0.55)	(−0.67 to −0.51)	(0.66 to 0.78)		
20 m sprint (s)	*r*	0.09	−0.20*	−0.16	−0.14	−0.69*	−0.66*	−0.77*	−0.72*	−0.67*	0.72*	0.94*	—
	(95% CI)	(−0.02 to 0.21)	(−0.31 to −0.09)	(−0.27 to −0.05)	(−0.25 to −0.02)	(−0.75 to −0.62)	(−0.72 to −0.58)	(−0.82 to −0.71)	(−0.77 to −0.66)	(−0.73 to −0.59)	(0.65 to 0.77)	(0.93 to 0.96)	

YBT-A: Y-balance test anterior distance; YBT-PM: Y-balance test posteromedial distance; YBT-PL: Y-balance test posterolateral distance; YBT-CS: Y-balance test composite score; DVJ: drop vertical jump; CMJ: countermovement jump; SLCMJ: single leg countermovement jump; SLJ: standing long jump; SLHD: single leg hop for distance.

* *p* < 0.05

Correlations in asymmetry between unilateral tests only demonstrated moderate-to-large relationships between the YBT-CS with the rest of the YBT measures (*r* = 0.46 to 0.69; *p* < 0.05), and between SLCMJ and SLHD (*r* = 0.32; *p* < 0.05) (Table 3).

**Table 3:** Correlations in interlimb asymmetries detected by unilateral tests.

**Test**		**YBT-A**	**YBT-PM**	**YBT-PL**	**YBT-CS**	**SLCMJ**	**SLHD**
YBT-A	*r*	—					
	(95% CI)						
YBT-PM	*r*	0.06	—				
	(95% CI)	(−0.05 to 0.17)					
YBT-PL	*r*	0.07	0.04	—			
	(95% CI)	(−0.04 to 0.18)	(−0.07 to 0.15)				
YBT-CS	*r*	0.46*	0.65*	0.69*	—		
	(95% CI)	(0.37 to 0.55)	(0.57 to 0.71)	(0.63 to 0.75)			
SLCMJ	*r*	0.02	0.12	0.05	0.11	—	
	(95% CI)	(−0.11 to 0.15)	(−0.01 to 0.24)	(−0.08 to 0.17)	(−0.02 to 0.23)		
SLHD	*r*	−0.02	0.09	−0.04	0.02	0.32*	—
	(95% CI)	(−0.13 to 0.10)	(−0.03 to 0.21)	(−0.16 to 0.07)	(−0.09 to 0.14)	(0.20 to 0.43)	

YBT-A: Y-balance test anterior distance; YBT-PM: Y-balance test posteromedial distance; YBT-PL: Y-balance test posterolateral distance; YBT-CS: Y-balance test composite score; SLCMJ: single leg countermovement jump; SLHD: single leg hop for distance.

* *p* < 0.05

The analysis of the correlation between asymmetry scores and test performances did not reveal any relevant association (all *r*-values < 0.29).

### 3.2 Differences by maturity status

Significant increases in jump scores and linear sprint and agility performances across maturation groups were found with medium to large ES (*p* < 0.01; *d* = 0.67–3.11) (Table 4). However, differences in the YBT performance were only found for the YBT-A and YBT-PM distances. Players in the post-PHV group reached significant greater YBT-PM distances than pre-PHV players with small ES (*p* = 0.03; *d* = 0.36). By contrast, pre-PHV reached greater YBT-A distance than circa- and post-PHV players with medium ES (*p* < 0.01; *d* = 0.54).

**Table 4:** Maturation-related differences for performance scores in the different tests.

Test	Maturity group			Whole group	Between maturity groups effects		Pairwise comparisons					
	Pre-PHV	Circa-PHV	Post-PHV				Pre-Circa		Pre-Post		Circa-Post	
	Mean ± SD	Mean ± SD	Mean ± SD	Mean ± SD	*p*-value	*ω* ^*2*^	*p*-value	*d*	*p*-value	*d*	*p*-value	*d*
YBT-A (%)	64.1 ± 5.4	61.3 ± 5.1	61.3 ± 5.1	62.7 ± 5.4	<0.01	0.06	0.01	0.54	<0.01	0.54	1.00	0.00
YBT-PM (%)	99.2 ± 8.9	99.6 ± 7.6	102.1 ± 6.9	100.3 ± 8.0	0.03	0.02	0.96	−0.05	0.03	−0.36	0.17	−0.31
YBT-PL (%)	95.7 ± 8.9	96.6 ± 7.7	96.9 ± 7.1	96.5 ± 8.0	0.50	0.00	1.00	−0.12	0.77	−0.16	1.00	−0.04
YBT-CS (%)	86.3 ± 6.8	85.9 ± 5.9	86.8 ± 5.1	86.5 ± 6.0	0.70	0.00	1.00	0.08	1.00	−0.07	1.00	−0.15
DVJ (cm)	22.4 ± 4.5	26.6 ± 4.3	30.9 ± 5.7	26.6 ± 6.0	<0.01	0.37	<0.01	−0.85	<0.01	−1.71	<0.01	−0.87
CMJ (cm)	24.0 ± 4.7	28.6 ± 3.6	31.8 ± 5.2	28.2 ± 5.7	<0.01	0.35	<0.01	−0.95	<0.01	−1.62	<0.01	−0.67
SLCMJ (cm)	10.3 ± 2.2	12.7 ± 2.9	17.1 ± 3.5	13.4 ± 4.1	<0.01	0.54	<0.01	−0.80	<0.01	−2.33	<0.01	−1.53
SLJ (cm)	156.0 ± 21.9	186.2 ± 17.5	205.0 ± 20.3	180.4 ± 29.0	<0.01	0.54	<0.01	−1.47	<0.01	−2.38	<0.01	−0.91
SLHD (%)	155.8 ± 22.6	176.3 ± 22.2	190.5 ± 18.0	173.5 ± 25.0	<0.01	0.36	<0.01	−0.99	<0.01	−1.67	<0.01	−0.68
Illinois agility (s)	17.7 ± 0.9	17.0 ± 1.1	16.2 ± 0.8	16.9 ± 1.1	<0.01	0.33	<0.01	0.75	<0.01	1.57	<0.01	0.82
10 m sprint (s)	2.1 ± 0.1	2.0 ± 0.1	1.9 ± 0.1	2.0 ± 0.2	<0.01	0.52	<0.01	1.32	<0.01	2.32	<0.01	0.99
20 m sprint (s)	3.7 ± 0.2	3.4 ± 0.2	3.2 ± 0.2	3.5 ± 0.3	<0.01	0.67	<0.01	1.85	<0.01	3.11	<0.01	1.26

YBT-A: Y-balance test anterior distance; YBT-PM: Y-balance test posteromedial distance; YBT-PL: Y-balance test posterolateral distance; YBT-CS: Y-balance test composite score; DVJ: drop vertical jump; CMJ: countermovement jump; SLCMJ: single leg countermovement jump; SLJ: standing long jump; SLHD: single leg hop for distance.

**Fig 1:**
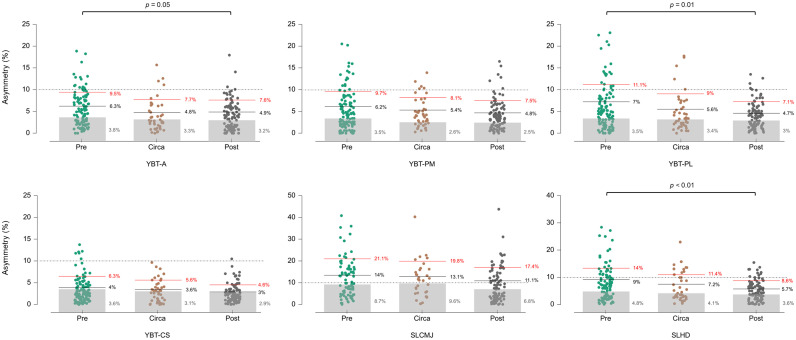
Individual asymmetry percentages for each unilateral test. Dashed lines separate individuals with asymmetry values above and below the proposed cut-off score of 10%. Black solid lines represent the population mean + smallest worthwhile change (SWC) (small to moderate) asymmetry threshold, red solid lines the population mean + SD (high) asymmetry threshold, and grey bars represent the mean within-subject coefficient of variation (CV%) for each test and maturity group according to Dos’Santos et al.’s [45] proposed calculations. *P*-values indicate differences in the proportion of players showing asymmetries ≥ 10% between maturation groups. YBT-A: Y-balance test anterior distance; YBT-PM: Y-balance test posteromedial distance; YBT-PL: Y-balance test posterolateral distance; YBT-CS: Y-balance test composite score; SLCMJ: single leg countermovement jump; SLHD: single leg hop for distance.

Significant reductions in mean interlimb asymmetry scores were found for the YBT-A and YBT-PL between pre- and post-PHV players with small ES (*p* < 0.03; *d* = 0.35–0.44). A significant reduction in mean asymmetry values between pre- and post-PHV players was also reported for the SLHD with medium ES (*p* < 0.01; *d* = 0.52) (Table 5). The individual analysis conducted for asymmetries showed a reduced proportion of players with asymmetries ≥10% for the YBT-A (14% vs. 4%; *p* = 0.05) and YBT-PL (18% vs. 5%; *p* = 0.01) from pre- to post-PHV groups, respectively; but the proportion of players with asymmetry values ≥10% for all the YBT measures remained low (≤ 18%) in all the maturation groups. This individual analysis also identified a decreased proportion of players with asymmetries ≥10% for the SLHD (28% vs. 10%; *p* < 0.01) from pre- to post-PHV groups, respectively. No differences in the proportion of players with asymmetries ≥10% for the SLCMJ were reported between pre-PHV (49%), circa-PHV (55%), and post-PHV (41%) groups (Fig 1).

**Table 5:** Maturation-related differences for interlimb asymmetries (%) in the unilateral tests.

Test	Maturity group			Whole group	Between maturity groups effects		Pairwise comparisons					
	Pre-PHV	Circa-PHV	Post-PHV				Pre-Circa		Pre-Post		Circa-Post	
	Mean ± SD	Mean ± SD	Mean ± SD	Mean ± SD	*p*-value	*ω* ^*2*^	*p*-value	*d*	*p*-value	*d*	*p*-value	*d*
YBT-A	5.5 ± 4.0	4.1 ± 3.6	4.2 ± 3.4	4.7 ± 3.7	0.02	0.02	0.10	0.38	0.03	0.35	1.00	−0.03
YBT-PM	5.3 ± 4.4	4.7 ± 3.4	4.1 ± 3.4	4.7 ± 3.8	0.06	0.01	0.58	0.17	0.07	0.31	0.64	0.14
YBT-PL	6.0 ± 5.1	4.7 ± 4.3	4.1 ± 3.0	5.1 ± 4.3	0.01	0.03	0.27	0.30	<0.01	0.44	0.67	0.15
YBT-CS	3.4 ± 2.9	3.1 ± 2.5	2.6 ± 2.0	3.0 ± 2.6	0.07	0.01	0.85	0.10	0.05	0.32	0.41	0.22
SLCMJ	12.2 ± 8.9	11.4 ± 8.4	9.5 ± 7.9	10.9 ± 8.3	0.10	0.01	1.00	0.10	0.10	0.33	0.78	0.23
SLHD	7.7 ± 6.3	6.1 ± 5.3	5.0 ± 3.6	6.6 ± 5.5	<0.01	0.05	0.26	0.31	<0.01	0.52	0.43	0.21

YBT-A: Y-balance test anterior distance; YBT-PM: Y-balance test posteromedial distance; YBT-PL: Y-balance test posterolateral distance; YBT-CS: Y-balance test composite score; SLCMJ: single leg countermovement jump; SLHD: single leg hop for distance.

## 4. Discussion

The main findings of this study indicated a moderate-to-very large relationship between all jump, sprint and agility performances. By contrast, weak correlations were reported between these tests and balance measures (with the only exception of SLHD). The highest relationships in performance scores were shown between tests evaluating similar movement patterns (e.g., linear sprint). The analysis of correlations for interlimb asymmetries did not report relevant relationships between different unilateral tests (*r* ≤0.32), and only asymmetries for the YBT-CS reached a moderate-to-large correlation with the rest of YBT measures. No meaningful association (*r* <0.29) was found between asymmetries and performance scores for any test. Performance in the different tests was clearly influenced by players’ maturity status. Although our results showed a significant reduction in asymmetry for YBT and SLHD tests from pre-PHV to post-PHV groups, a limited impact of maturation on the magnitude of asymmetries was evident.

Traditionally, testing batteries have been used to monitor athletes’ performance, identify talent, and determine injury risk, or indicate readiness to return to training or competition after an injury [3, 4, 14]. Many different tests have been proposed for these purposes [3, 4] and, probably, more will emerge in the future. Thus, testing batteries with the best prognostic/diagnostic and the shortest time commitment are essential and therefore, including multiple tests that have a large common variance may be counterproductive to this aim [14]. Interestingly, our study showed moderate-to-very-large relationships between jump, sprint, and agility performances in adolescent soccer players. These findings are in line with our hypothesis and previous research in soccer [7, 17], and suggest that individuals with better jump performance tend to be faster (i.e., less time) in multidirectional speed tests. The necessity for maximal relative impulse to be produced for acceleration in each of these tests may explain these relationships [46, 47]. However, it should be noted that, excluding the correlation between both linear sprint tests (*r* = 0.94), the shared variance between the rest of the jump and multidirectional sprint tests was less than 66%, even for tests assessing the same movement patterns (e.g., SLJ vs. SLHD). Thus, while multidirectional sprint performance may also be enhanced by specific training interventions for jump ability (and vice versa), our results suggest that these tests are measuring different qualities and should not be used interchangeably. Strength and conditioning coaches need, therefore, to select tests that have the greatest specificity for the activity of interest and sport when designing an efficient screening battery.

As hypothesized, an influence of maturation on players’ physical performance was also revealed in our study, with individuals increasing their jump, sprint and agility scores from pre- to circa- and post-PHV groups. These findings match with previous results in male youth soccer [31, 32], and may reflect a natural improvement in the ability to perform stretch-shortening cycle (SSC) tasks with maturation [48]. Although the underlying mechanisms were not explored in our study, it has been suggested that increments in muscle size, pennation angle, fascicle length, tendon stiffness, motor-unit recruitment and preactivation, as well as reduced muscle co-contraction could be behind the enhancement of force production characteristics and SSC function during maturational years [48]. Previous researchers have also suggested that balance capabilities would be compromised in children and, as they mature, an improvement in postural control might be expected [49, 50]. This improvement in postural control has been linked to both body physical changes (configurations and proportions) and sensoriomotor control development (learning to calibrate and integrate visual, vestibular, and somatosensory information) [33, 50]. However, our analysis by maturity status on balance performance only support partially these suggestions, as improvements from pre- to post-PHV players were only identified for the YBT-PM direction (small ES). Moreover, players also showed a moderate (*d* = 0.54) reduction in performance on this test from pre-PHV to circa- and post-PHV groups for the YBT-A distance, which is also in line with earlier research [33]. Two main reasons may explain the small increase, or even decrease, in balance performance with advancing maturational stage found in our study. On the one hand, YBT scores were normalized and presented relative to leg length, which may have reduced the impact of anthropometric changes as players mature. On the other hand, a very large relationship between YBT-A distance performance and ankle dorsiflexion has previously been found [51] and as such, performance on this test may be determined more by ankle mobility as opposed to dynamic balance [33, 34].

It seems reasonable to think that the observed differences between maturational groups for physical performances may also affect the correlations between tests. In fact, previous researchers have stated that during the early stages of maturation, basic improvements in one component (e.g., speed) may also enhance performance within another (e.g., agility) [38]. To check this, additional correlation analyses by maturation group were carried out in our study (see S1 Table). These results confirm an influence of maturation on the strength of the correlations achieved between tests, with pre-PHV generally showing stronger correlations than circa- and, especially, post-PHV players. The trend in these relationships was, however, similar across maturity states, with the highest correlations being found between tests evaluating similar movement patterns.

When examining the relationships between asymmetries across different unilateral tests, our results also support the notion that interlimb asymmetries are task-dependent, as previously described [20, 21, 25]. Indeed, the only noteworthy correlation for asymmetries between tests was that of the YBT-CS with the rest of YBT measures (*r* = 0.46–0.69), which is unsurprising as the YBT-CS is a measure obtained from the average of the three directions (anterior, posteromedial, and posterolateral) assessed in the YBT. Likewise, our additional analyses revealed a low (≤6.6%) mean asymmetry score for almost all measures, with the only exception of the SLCMJ (10.9%), and a lack of influence of asymmetry on test performances (*r* <0.29). Cumulatively, these findings might lead coaches to dispense with the analysis of asymmetries in their proposals for testing batteries, as the cost-benefit ratio of this screening would be questionable (i.e., need for multiple tests vs. lack of relevance to performance). However, our asymmetry data should be interpreted with caution, as they are the result of a single testing session and may not reflect the true picture of the presence of asymmetries compared to studies using repeated time point analyses or testing sessions [52]. Given that other recent studies do show associations between interlimb asymmetries (assessed through similar tests) and physical performance [18, 20, 52], more research is still needed to clarify this issue. Furthermore, the importance of asymmetries is not only related to performance: previous researchers have found an increased likelihood of injury among youth athletes with asymmetries ≥10% [27, 28]. In this regard, the individual analysis conducted in our study has shown a considerable proportion of total players with asymmetries ≥10% for the SLHD (~20%) and, especially, for the SLCMJ (~50%). Moreover, unlike what was found for test performance, asymmetries were not strongly affected by the maturational development of the players (no differences for the SLCMJ and limited ES ≤0.5 for the SLHD, and for the YBT as well), so those players exhibiting large asymmetries during the pre-PHV stage could be expected to maintain these interlimb differences as they mature. Therefore, the high prevalence, the potential relationship to injury risk, and the unchanging nature with maturation mean that the assessment of asymmetries (and specific intervention when detected) should remain as a primary objective of testing batteries. The SLCMJ has proven to be the most sensitive test to identify large asymmetries in our cohort of male youth soccer players. However, it should be noted that asymmetry values are task- and metric-specific [53], so practitioners should also consider the use of specific thresholds to their athlete population, metrics, and tests that assist in the interpretation and classification of interlimb asymmetry scores. The calculations proposed by Dos’Santos et al.’s [45] might be viewed as a good option for this purpose.

In the current study we used a comprehensive approach to concurrently explore both the correlation between tests assessing different physical performance abilities (i.e., jump, balance, multidirectional sprint) and the interrelationships between tests (e.g., DVJ, CMJ, SLCMJ) involving the same movement pattern (i.e., vertical jump) in a large cohort of male youth soccer players. This in-depth analysis also included the exploration of relationships between interlimb asymmetries across unilateral tests, taking into consideration the interplayer variability in asymmetry values. Additionally, potential changes in performance and asymmetry scores throughout adolescence were examined according to the players’ maturity status, rather than analyses based on chronological age. Despite these strengths, our study also presents some limitations. Firstly, only outcome measures (i.e., height/distance for jumps, time for sprints) were analyzed. Although these are the most commonly used metrics in applied settings, some researchers [54] have recently recommended the inclusion of specific force-time variables when profiling athletes’ physical performance in these tasks. Secondly, specific tests of muscle strength were not included in this research. There is extensive evidence showing the importance of strength for sport performance and injury risk reduction in youth athletes [55], as well as its influence on other performance abilities [16]. The inclusion of this component is therefore key when testing youth athletes. Finally, the maturity status was calculated using a regression equation, which may not be as accurate as skeletal imaging; however, to minimize the group allocation error derived from this equation, players with a maturational offset between -1 and -0.5 and 0.5 to 1 were removed from the data set when running these analyses.

## 5. Conclusions

A moderate-to-very large relationship between all jump, sprint and agility performances was found in this study, but the shared variance between almost all these tests was less than 66% (with the only exception of both 10 m and 20 m linear sprints [*R*^2^ = 88%]). These findings indicate that these tests would be evaluating different physical abilities and should not be used interchangeably. However, the high correlations found between these tests (especially between those evaluating similar movement patterns) seem to indicate a similar sensitivity to training and thus, coaches might prioritize those with the greatest specificity for their sport. Due to its influence on players’ physical performance, it is important for coaches to take growth and maturation into account when comparing and interpreting their players’ results in these tests.

Our results also showed that interlimb asymmetries vary across different unilateral tests, and that these side-to-side differences might not affect players’ performance on balance, jumps, and multidirectional sprints. However, a great percentage (~50%) of total players reported large (≥10%) asymmetry scores for the SLCMJ. These asymmetries do not seem to be largely reduced with the natural advance in maturation. Therefore, and given their potential relationships to injury risk, analysis of interlimb asymmetries should remain as a major component of the testing batteries implemented by coaches. The SLCMJ might be viewed as an appropriate test to do this in male youth soccer.

## Supporting information

S1 Table Pearson’s correlations (*r*) by maturation group. (DOCX)

## References

[1] Bangsbo J, Mohr M, Krustrup P. Physical and metabolic demands of training and match-play in the elite football player. J Sports Sci. 2006;24(7):665–74. doi: 10.1080/02640410500482529 16766496

[2] Faude O, Koch T, Meyer T. Straight sprinting is the most frequent action in goal situations in professional football. J Sports Sci. 2012;30(7):625–31. doi: 10.1080/02640414.2012.665940 22394328

[3] Read PJ, Oliver JL, De Ste Croix MBA, Myer GD, Lloyd RS. A review of field-based assessments of neuromuscular control and their utility in male youth soccer players. J Strength Cond Res. 2019;33(1):283–99. doi: 10.1519/JSC.0000000000002069 28658071 PMC6604066

[4] Comfort P, Jones PA, McMahon JJ. Performance assessment in strength and conditioning. Abingdon: Routledge; 2018.

[5] Cronin JB, Hansen KT. Strength and power predictors of sports speed. J Strength Cond Res. 2005;19(2):349–57. doi: 10.1519/14323.1 15903374

[6] McFarland IT, Dawes JJ, Elder CL, Lockie RG. Relationship of two vertical jumping tests to sprint and change of direction speed among male and female collegiate soccer players. Sports. 2016;4(1):1–7. doi: 10.3390/sports4010011 29910258 PMC5968930

[7] Sonesson S, Lindblom H, Hägglund M. Performance on sprint, agility and jump tests have moderate to strong correlations in youth football players but performance tests are weakly correlated to neuromuscular control tests. Knee Surgery, Sport Traumatol Arthrosc. 2021;29(5):1659–69. doi: 10.1007/s00167-020-06302-z 33030610 PMC8038985

[8] Vescovi JD, Mcguigan MR. Relationships between sprinting, agility, and jump ability in female athletes. J Sports Sci. 2008;26(1):97–107. doi: 10.1080/02640410701348644 17852692

[9] Wisløff U, Castagna C, Helgerud J, Jones R, Hoff J. Strong correlation of maximal squat strength with sprint performance and vertical jump height in elite soccer players. Br J Sports Med. 2004;38(3):285–8. doi: 10.1136/bjsm.2002.002071 15155427 PMC1724821

[10] Chamari K, Hachana Y, Ahmed YB, Galy O, Sghaïer F, Chatard J-C, et al. Field and laboratory testing in young elite soccer players. Br J Sports Med. 2004;38(2):191–6. doi: 10.1136/bjsm.2002.004374 15039258 PMC1724764

[11] Chelly MS, Chérif N, Amar M Ben, Hermassi S, Fathloun M, Bouhlel E, et al. Relationships of peak leg power, 1 maximal repetition half back squat, and leg muscle volume to 5-m sprint performance of junior soccer players. J Strength Cond Res. 2010;24(1):266–71. doi: 10.1519/JSC.0b013e3181c3b298 19924009

[12] Kukolj M, Ropret R, Ugarkovic D, Jaric S. Anthropometric, strength, and power predictors of sprinting performance. J Sports Med Phys Fitness. 1999;39(2):120–2. 10399419

[13] Salaj S, Markovic G. Specificity of jumping, sprinting, and quick change-of-direction motor abilities. J Strength Cond Res. 2011;25(5). doi: 10.1519/JSC.0b013e3181da77df 21240031

[14] Meylan C, McMaster T, Cronin J, Mohammad NI, Rogers C, DeKlerk M. Single-leg lateral, horizontal, and vertical jump assessment: reliability, interrelationships, and ability to predict sprint and change-of-direction performance. J Strength Cond Res. 2009;23(4):1140–7. doi: 10.1519/JSC.0b013e318190f9c2 19528866

[15] Köklü Y, Alemdaroğlu U, Özkan A, Koz M, Ersöz G. The relationship between sprint ability, agility and vertical jump performance in young soccer players. Sci Sports. 2015;30(1):e1–5.

[16] Comfort P, Stewart A, Bloom L, Clarkson B. Relationships between strength, sprint, and jump performance in well-trained youth soccer players. J Strength Cond Res. 2014;28(1):173–7. doi: 10.1519/JSC.0b013e318291b8c7 23542878

[17] Parr J, Winwood K, Hodson-Tole E, Deconinck FJA, Hill JP, Teunissen JW, et al. The main and interactive effects of biological maturity and relative age on physical performance in elite youth soccer players. J Sports Med. 2020;2020:1–11.

[18] Bishop C, Brashill C, Abbott W, Read P, Lake J, Turner A. Jumping asymmetries are associated with speed, change of direction speed, and jump performance in elite academy soccer players. J Strength Cond Res. 2021;35(7):1841–7. doi: 10.1519/JSC.0000000000003058 30707141

[19] Read PJ, Oliver JL, Myer GD, De Ste Croix MBA, Lloyd RS. The effects of maturation on measures of asymmetry during neuromuscular control tests in elite male youth soccer players. Pediatr Exerc Sci. 2018;30(1):168–75. doi: 10.1123/pes.2017-0081 28787266 PMC6538932

[20] Bishop C, Read PJ, McCubbine J, Turner A. Vertical and horizontal asymmetries are related to slower sprinting and jump performance in elite youth female soccer players. J Strength Cond Res. 2021;35(1):56–63. doi: 10.1519/JSC.0000000000002544 29489719

[21] Fox KT, Pearson LT, Hicks KM. The effect of lower inter-limb asymmetries on athletic performance: a systematic review and meta-analysis. PLoS One. 2023;18(6):e0286942. doi: 10.1371/journal.pone.0286942 37289826 PMC10249853

[22] Bishop C, Abbott W, Brashill C, Turner A, Lake J, Read P. Bilateral vs. unilateral countermovement jumps: comparing the magnitude and direction of asymmetry in elite academy soccer players. J Strength Cond Res. 2022;36(6):1660–6. doi: 10.1519/JSC.0000000000003679 35622111

[23] Bishop C, Read P, Chavda S, Jarvis P, Brazier J, Bromley T, et al. Magnitude or direction? Seasonal variation of interlimb asymmetry in elite academy soccer players. J Strength Cond Res. 2022;36(4):1031–7. doi: 10.1519/JSC.0000000000003565 32149878

[24] Bishop C, Read P, Stern D, Turner A. Effects of soccer match-play on unilateral jumping and interlimb asymmetry: a repeated-measures design. J Strength Cond Res. 2022;36(1):193–200. doi: 10.1519/JSC.0000000000003389 31985557

[25] Bishop C, Lake J, Loturco I, Papadopoulos K, Turner A, Read P. Interlimb asymmetries: the need for an individual approach to data analysis. J Strength Cond Res. 2021;35(3):695–701. doi: 10.1519/JSC.0000000000002729 33587548

[26] Hart NH, Nimphius S, Weber J, Spiteri T, Rantalainen T, Dobbin M, et al. Musculoskeletal asymmetry in football athletes: a product of limb function over time. Med Sci Sports Exerc. 2016;48(7):1379–87. doi: 10.1249/MSS.0000000000000897 26871989

[27] Oliver JL, Ayala F, De Ste Croix MBA, Lloyd RS, Myer GD, Read PJ. Using machine learning to improve our understanding of injury risk and prediction in elite male youth football players. J Sci Med Sport. 2020;23(11):1044–8. doi: 10.1016/j.jsams.2020.04.021 32482610

[28] Fort-Vanmeerhaeghe A, Milà-Villarroel R, Pujol-Marzo M, Arboix-Alió J, Bishop C. Higher vertical jumping asymmetries and lower physical performance are indicators of increased injury incidence in youth team-sport athletes. J Strength Cond Res. 2022;36(8):2204–11. doi: 10.1519/JSC.0000000000003828 33009354

[29] Lockie RG, Callaghan SJ, Berry SP, Cooke ERA, Jordan CA, Luczo TM, et al. Relationship between unilateral jumping ability and asymmetry on multidirectional speed in team-sport athletes. J Strength Cond Res. 2014;28(12):3557–66. doi: 10.1519/JSC.0000000000000588 24942166

[30] Lloyd RS, Oliver JL, Hughes MG, Williams CA. The influence of chronological age on periods of accelerated adaptation of stretch-shortening cycle performance in pre and postpubescent boys. J Strength Cond Res. 2011;25(7):1889–97. doi: 10.1519/JSC.0b013e3181e7faa8 21499135

[31] Selmi MA, Sassi RH, Yahmed MH, Giannini S, Perroni F, Elloumi M. Normative data and physical determinants of multiple sprint sets in young soccer players aged 11–18 years: effect of maturity status. J Strength Cond Res. 2020;34(2):506–15. doi: 10.1519/JSC.0000000000002810 30239457

[32] Read PJ, Oliver JL, De Ste Croix MBA, Myer GD, Lloyd RS. Hopping and landing performance in male youth soccer players: effects of age and maturation. Int J Sports Med. 2017;38(12):902–8. doi: 10.1055/s-0043-114009 28931173

[33] Read PJ, Oliver JL, Myer GD, Farooq A, De Ste Croix M, Lloyd RS. Utility of the anterior reach Y-Balance test as an injury risk screening tool in elite male youth soccer players. Phys Ther Sport. 2020;45:103–10. doi: 10.1016/j.ptsp.2020.06.002 32726731 PMC9892799

[34] Holden S, Boreham C, Doherty C, Wang D, Delahunt E. A longitudinal investigation into the progression of dynamic postural stability performance in adolescents. Gait Posture. 2016;48:171–6. doi: 10.1016/j.gaitpost.2016.04.019 27285476

[35] Asimakidis ND, Dalamitros AA, Ribeiro J, Lola AC, Manou V. Maturation stage does not affect change of direction asymmetries in young soccer players. J Strength Cond Res. 2022;36:3440–5. doi: 10.1519/JSC.0000000000004110 36417358

[36] Taylor KL, Sheppard JM, Lee H, Plummer N. Negative effect of static stretching restored when combined with a sport specific warm-up component. J Sci Med Sport. 2009;12(6):657–61. doi: 10.1016/j.jsams.2008.04.004 18768355

[37] Mirwald RL, Baxter-Jones ADG, Bailey DA, Beunen GP. An assessment of maturity from anthropometric measurements. Med Sci Sports Exerc. 2002;34(4):689–94. doi: 10.1097/00005768-200204000-00020 11932580

[38] Paul DJ, Nassis GP. Physical fitness testing in youth soccer: issues and considerations regarding reliability, validity and sensitivity. Pediatr Exerc Sci. 2015;27(3):301–13. doi: 10.1123/mc.2014-0085 26331619

[39] Shaffer SW, Teyhen DS, Lorenson CL, Warren RL, Koreerat CM, Straseske CA, et al. Y-balance test: a reliability study involving multiple raters. Mil Med. 2013;178(11):1264–70. doi: 10.7205/MILMED-D-13-00222 24183777

[40] Thomas K, French D, Hayes PR. The effect of two plyometric training techniques on muscular power and agility in youth soccer players. J Strength Cond Res. 2009;23(1):332–5. doi: 10.1519/JSC.0b013e318183a01a 19002073

[41] Hopkins WG. A new view of statistics: a scale of magnitudes for effect statistics. [Internet]. 2002 [cited 2023 Jun 5]. https://www.sportsci.org/resource/stats/effectmag.html

[42] Field A. Discovering statistics using IBM SPSS statistics (4th edition). London: SAGE Publications Ltd; 2013.

[43] Cohen J. Statistical power analysis for the behavioral sciences (2nd ed.). New Jersey: Lawrence Erlbaum Associates; 1988.

[44] Bishop C, Read P, Lake J, Chavda S, Turner A. Interlimb asymmetries: understanding how to calculate differences from bilateral and unilateral tests. Strength Cond J. 2018;40(4):1–6.

[45] Dos’Santos T, Thomas C, Jones PA. Assessing interlimb asymmetries: are we heading in the right direction? Strength Cond J. 2021;43(3):91–100.

[46] Kirby TJ, McBride JM, Haines TL, Dayne AM. Relative net vertical impulse determines jumping performance. J Appl Biomech. 2011;27(3):207–14. doi: 10.1123/jab.27.3.207 21844609

[47] Hunter JP, Marshall RN, McNair PJ. Relationships between ground reaction force impulse and kinematics of sprint-running acceleration. J Appl Biomech. 2005;21(1):31–43. doi: 10.1123/jab.21.1.31 16131703

[48] Radnor JM, Oliver JL, Waugh CM, Myer GD, Moore IS, Lloyd RS. The influence of growth and maturation on stretch-shortening cycle function in youth. Sports Med. 2018;48(1):57–71. doi: 10.1007/s40279-017-0785-0 28900862 PMC5752749

[49] Pau M, Arippa F, Leban B, Corona F, Ibba G, Todde F, et al. Relationship between static and dynamic balance abilities in Italian professional and youth league soccer players. Phys Ther Sport. 2015;16(3):236–41. doi: 10.1016/j.ptsp.2014.12.003 25869425

[50] Riach CL, Starkes JL. Velocity of centre of pressure excursions as an indicator of postural control systems in children. Gait Posture. 1994;2(3):167–72.

[51] Overmoyer G V, Reiser RFII. Relationships between lower-extremity flexibility, asymmetries, and the Y balance test. J Strength Cond Res. 2015;29(5):1240–7. doi: 10.1519/JSC.0000000000000693 25719917

[52] Bishop C, Perez-Higueras Rubio M, Gullon IL, Maloney S, Balsalobre-Fernandez C. Jump and change of direction speed asymmetry using smartphone apps: between-session consistency and associations with physical performance. J Strength Cond Res. 2022;36(4):927–34. doi: 10.1519/JSC.0000000000003567 32149875

[53] Bishop C. Interlimb asymmetries: are thresholds a usable concept? Strength Cond J. 2021;43(1):32–6.

[54] Bishop C, Turner A, Jordan M, Harry J, Loturco I, Lake J, et al. A framework to guide practitioners for selecting metrics during the countermovement and drop jump tests. Strength Cond J. 2022;44(4):95–103.

[55] Chaabene H, Lesinski M, Behm DG, Granacher U. Performance—and health-related benefits of youth resistance training. Sport Orthop Traumatol. 2020;36(3):231–40.

