# Supplementary material for: Things to keep in mind when selecting physical assessments in youth soccer: Correlations between test performances, interlimb asymmetries, and effects of maturation
Source: PLoS One. 2024 Jun 21;19(6):e0305570. doi: 10.1371/journal.pone.0305570 (PMC11192305; doi:10.1371/journal.pone.0305570)
Supplement: S1 Table — (DOCX) [file pone.0305570.s001.docx]

S1 Table. Pearson's correlations (*r*) by maturation group.

| **Test** |  | **YBT-A (%)** | **YBT-PM (%)** | **YBT-PL (%)** | **YBT-CS (%)** | **DVJ (cm)** | **CMJ (cm)** | **SLCMJ (cm)** | **SLJ (cm)** | **SLHD (cm)** | **Illinois agility (s)** | **10 m sprint (s)** | **20 m sprint (s)** |
| --- | --- | --- | --- | --- | --- | --- | --- | --- | --- | --- | --- | --- | --- |
| YBT-A (%) | Pre-PHV | **—** |  |  |  |  |  |  |  |  |  |  |  |
|  | Circa-PHV |  |  |  |  |  |  |  |  |  |  |  |  |
|  | Post-PHV |  |  |  |  |  |  |  |  |  |  |  |  |
| YBT-PM (%) | Pre-PHV | 0.54* | — |  |  |  |  |  |  |  |  |  |  |
|  | Circa-PHV | 0.46* |  |  |  |  |  |  |  |  |  |  |  |
|  | Post-PHV | 0.35* |  |  |  |  |  |  |  |  |  |  |  |
| YBT-PL (%) | Pre-PHV | 0.60* | 0.74* | — |  |  |  |  |  |  |  |  |  |
|  | Circa-PHV | 0.59* | 0.75* |  |  |  |  |  |  |  |  |  |  |
|  | Post-PHV | 0.24 | 0.65* |  |  |  |  |  |  |  |  |  |  |
| YBT-CS (%) | Pre-PHV | 0.76* | 0.90* | 0.92* | — |  |  |  |  |  |  |  |  |
|  | Circa-PHV | 0.74* | 0.89* | 0.93* |  |  |  |  |  |  |  |  |  |
|  | Post-PHV | 0.61* | 0.88* | 0.85* |  |  |  |  |  |  |  |  |  |
| DVJ (cm) | Pre-PHV | 0.05 | 0.16 | 0.12 | 0.14 | — |  |  |  |  |  |  |  |
|  | Circa-PHV | 0.17 | −0.17 | −0.17 | −0.10 |  |  |  |  |  |  |  |  |
|  | Post-PHV | 0.13 | 0.13 | 0.01 | 0.11 |  |  |  |  |  |  |  |  |
| CMJ (cm) | Pre-PHV | 0.08 | 0.25 | 0.32* | 0.28 | 0.77* | — |  |  |  |  |  |  |
|  | Circa-PHV | 0.03 | −0.26 | −0.15 | −0.17 | 0.60* |  |  |  |  |  |  |  |
|  | Post-PHV | 0.21 | 0.08 | 0.03 | 0.12 | 0.65* |  |  |  |  |  |  |  |
| SLCMJ (cm) | Pre-PHV | 0.08 | 0.17 | 0.19 | 0.18 | 0.57* | 0.73* | — |  |  |  |  |  |
|  | Circa-PHV | 0.33 | 0.00 | 0.17 | 0.17 | 0.54* | 0.52 |  |  |  |  |  |  |
|  | Post-PHV | 0.05 | 0.15 | 0.17 | 0.16 | 0.47* | 0.70* |  |  |  |  |  |  |
| SLJ (cm) | Pre-PHV | 0.26 | 0.35* | 0.39* | 0.39* | 0.26 | 0.35* | 0.31 | — |  |  |  |  |
|  | Circa-PHV | 0.28 | 0.20 | 0.08 | 0.20 | 0.33 | 0.28 | 0.41 |  |  |  |  |  |
|  | Post-PHV | 0.08 | 0.25 | 0.20 | 0.24 | 0.32* | 0.51* | 0.48* |  |  |  |  |  |
| SLHD (cm) | Pre-PHV | 0.27 | 0.49* | 0.42* | 0.47* | 0.28 | 0.37* | 0.30 | 0.77* | — |  |  |  |
|  | Circa-PHV | 0.34 | 0.32 | 0.42 | 0.42 | 0.37 | 0.25 | 0.58* | 0.55* |  |  |  |  |
|  | Post-PHV | −0.01 | 0.31* | 0.25 | 0.25 | 0.32* | 0.36* | 0.48* | 0.68* |  |  |  |  |
| Illinois agility (s) | Pre-PHV | −0.38* | −0.36* | −0.34* | −0.40* | −0.26 | −0.36* | −0.48* | −0.42* | −0.35* | — |  |  |
|  | Circa-PHV | −0.27 | −0.24 | −0.41 | −0.36 | −0.28 | −0.13 | −0.28 | 0.06 | −0.45* |  |  |  |
|  | Post-PHV | −0.13 | −0.15 | −0.12 | −0.17 | 0.00 | −0.09 | −0.15 | −0.23 | −0.30* |  |  |  |
| 10 m sprint (s) | Pre-PHV | −0.22 | −0.14 | −0.17 | −0.20 | −0.46* | −0.57* | −0.48* | −0.32* | −0.31* | 0.46* | — |  |
|  | Circa-PHV | −0.22 | −0.08 | −0.18 | −0.17 | −0.34 | −0.19 | −0.40 | −0.25 | −0.45* | 0.67* |  |  |
|  | Post-PHV | 0.01 | −0.01 | −0.09 | −0.05 | −0.06 | −0.04 | −0.38* | −0.14 | −0.25 | 0.51* |  |  |
| 20 m sprint (s) | Pre-PHV | −0.25 | −0.20 | −0.22 | −0.26 | −0.50* | −0.59* | −0.51* | −0.41* | −0.38* | 0.53* | 0.93* | — |
|  | Circa-PHV | −0.18 | 0.02 | −0.07 | −0.07 | −0.46* | −0.30 | −0.53* | −0.31 | −0.46* | 0.63* | 0.92* |  |
|  | Post-PHV | 0.04 | −0.07 | −0.12 | −0.07 | −0.29 | −0.21 | −0.46* | −0.30* | −0.39* | 0.51* | 0.87* |  |
| YBT-A: Y-balance test anterior distance; YBT-PM: Y-balance test posteromedial distance; YBT-PL: Y-balance test posterolateral distance; YBT-CS: Y-balance test composite score; DVJ: drop vertical jump; CMJ: countermovement jump; SLCMJ: single leg countermovement jump; SLJ: standing long jump; SLHD: single leg hop for distance.  * *p* < 0.05 | | | | | | | | | | | | | |
